# AMA‐Positive Cholestasis in Celiac Disease: Why Primary Biliary Cholangitis Should Not Be Excluded by Biopsy Alone

**DOI:** 10.1002/ccr3.72954

**Published:** 2026-06-14

**Authors:** Muhammad Abdullah Awan

**Affiliations:** ^1^ Wah Medical College National University of Medical Sciences Pakistan

**Keywords:** antimitochondrial antibodies, autoimmune liver disease, celiac disease, cholestasis, liver biopsy, primary biliary cholangitis

## Abstract

In celiac disease patients with AMA‐M2 positivity, cholestatic enzymes, pruritus, and fibrosis, early primary biliary cholangitis should remain under longitudinal surveillance; nondiagnostic biopsy alone should not exclude autoimmune cholestatic overlap.

Dear Editor,

I read with great interest the case report by Hussain et al., describing constipation with anti‐mitochondrial antibody (AMA) and antinuclear antibody (ANA) positivity as an atypical manifestation of celiac disease [1]. The report is clinically valuable because it reminds physicians that celiac disease may present beyond the classic diarrheal phenotype and may be accompanied by extraintestinal hepatobiliary abnormalities.

However, one diagnostic aspect deserves further clarification. In the reported patient, the coexistence of raised alkaline phosphatase and gamma‐glutamyl transferase, pruritus, AMA‐M2 positivity, ANA positivity, and F2 fibrosis suggests a cholestatic autoimmune liver phenotype that overlaps with the diagnostic territory of primary biliary cholangitis (PBC) [1]. Although the authors favored celiac hepatitis and noted that liver biopsy did not support PBC, exclusion of early PBC on biopsy alone may be premature.

This distinction is important because PBC can be patchy in distribution, especially in early disease, and a nondiagnostic biopsy does not necessarily exclude it when serology and cholestatic biochemistry are suggestive. In such cases, the diagnostic weight should not rest on histology alone. Serial alkaline phosphatase and gamma‐glutamyl transferase trends, immunoglobulin M levels, AMA titers, adherence to a gluten‐free diet, and biochemical response over follow‐up would help determine whether the liver injury behaves like isolated celiac‐associated hepatitis or persistent autoimmune cholestasis.

Another important point is therapeutic interpretation. Improvement in transaminases after a gluten‐free diet may support celiac‐related liver involvement, but persistent cholestatic enzymes despite dietary adherence would warrant continued hepatology follow‐up and reconsideration of PBC‐directed management. Therefore, this case may be best framed as celiac disease with AMA‐positive cholestatic liver injury requiring longitudinal classification, rather than as celiac hepatitis alone at initial presentation.

## 1 Conclusion

Hussain et al. present an important atypical case of celiac disease. Nevertheless, AMA‐M2 positivity with cholestatic liver enzymes, pruritus, and fibrosis should prompt continued surveillance for early PBC or autoimmune cholestatic overlap, even when liver biopsy is nondiagnostic. This approach would improve diagnostic precision and guide safer long‐term follow‐up.

Sincerely,

## Author Contributions

**Muhammad Abdullah Awan:** conceptualization, formal analysis, writing – original draft, writing – review and editing.

## Funding

The author has nothing to report.

## Ethics Statement

The author has nothing to report.

## Consent

The author has nothing to report.

## Conflicts of Interest

The author declares no conflicts of interest.

## Data Availability

No new data were generated or analyzed for this letter.
